# 

**Published:** 1857-10

**Authors:** 


*New Method of treating Abscess.*—We find under this heading the following in the New Orleans Medical News and Hospital Gazette:

"In the July number of the Southern Medical and Surgical Journal, we notice interesting extracts from letters from Dr. T. A. Means, of Georgia, now in Paris. He says: 'M. Chassaignac has now in his hospital some forty patients under treatment for *abscess*, by a new system which he terms *drainage*. It is simple, rational, and effective. He employs a gum elastic tube about one-eighth of an inch in diameter, perforated along its sides with small holes, at intervals or two or three inches. When called to operate, he thrusts a trocar and canula entirely through the abscess at its base—withdraws the trocar, inserts the tube in the canula, passes it through, and then removes the canula itself—leaving only the flexible tube to perform its work of drainage. All can be done in ten minutes, or less time. Some two or three times per day, the tube should be moved, one or two inches, so as to allow a free exit of pus through the openings.'"

---

"*The Retired Physician.*"—The readers of the newspapers for the last few months, must have noticed an announcement of the existence of a "retired physician whose sands of life have nearly run out," hailing from Jersey city. This aged advertiser of a quack nostrum is said to be a young man about twenty-five years old, in good health, and engaged most of the time in writing for the New York Sunday papers. Such is the inexhaustable credulity of a portion of mankind on the subject of remedies, that we presume this "new dodge" has proved remunerative to the inventor.

